# A prospective longitudinal analysis of the predictors of amenorrhea after breast cancer chemotherapy: Impact of *BRCA* pathogenic variants

**DOI:** 10.1002/cam4.6527

**Published:** 2023-09-12

**Authors:** Kutluk H. Oktay, Volkan Turan, Giuliano Bedoschi, Nadia Abdo, Heejung Bang, Shari Goldfarb

**Affiliations:** ^1^ Department of Obstetrics and Gynecology and Reproductive Sciences Yale University School of Medicine New Haven Connecticut USA; ^2^ Department of Obstetrics and Gynecology Istanbul Health and Technology University School of Medicine Istanbul Turkey; ^3^ Department of Obstetrics and Gynecology, Ribeirao Preto School of Medicine University of Sao Paulo Ribeirao Preto Brazil; ^4^ Memorial Sloan Kettering Cancer Center New York New York USA; ^5^ Division of Biostatistics, Department of Public Health Sciences University of California Davis California USA; ^6^ Weill Cornell Medical Center New York New York USA

**Keywords:** amenorrhea, anti‐mullerian hormone, *BRCA*, breast cancer, chemotherapy, fertility preservation

## Abstract

**Background:**

Better tools for post‐chemotherapy amenorrhea risk assessment are needed for fertility preservation decision‐making. Our aim was to determine the predictors of amenorrhea risk at 12 and 18 months post‐chemotherapy in women with breast cancer.

**Methods:**

142 women with breast cancer were longitudinally followed for their menstrual changes at 6, 12, and 18 months after the completion of adjuvant chemotherapy with an Anthracycline‐Cyclophosphamide‐based (AC‐based) or Cyclophosphamide‐Methotrexate +5‐Fluorouracil regimen. Pre‐ and/or post‐chemo AMH levels, age, BMI, tamoxifen use, regimen type, and germline *BRCA* pathogenic variant (*gBRCApv*) status were evaluated for the prediction of amenorrhea at 6–18 months.

**Results:**

In multivariable‐adjusted logistic regression, age (*p* = 0.03) and AMH (*p* = 0.03) at 12 months, and *gBRCApv* status (*p* = 0.03) at 18 months were significant predictors of amenorrhea (areas under the ROC curve of 0.77 and 0.76, for 12 and 18 months, respectively) among 102 evaluable subjects. An undetectable AMH immediately post‐chemotherapy was predictive of amenorrhea with <18 month follow‐up. In longitudinal analysis estimating time trends, baseline AMH and *gBRCApv* status was associated with the risk of amenorrhea over 6–18 months; the AMH >2.0 ng/mL group showed attenuated time‐trend risk of amenorrhea versus AMH ≤2.0 group (ratio of ORs = 0.91, 95% CI = 0.86–0.97, *p* = 0.002), while the *gBRCApv* + showed a steeper time trend, versus the controls (ratio of ORs = 1.12, 95% CI = 1.04–1.20, *p* = 0.003).

**Conclusions:**

In addition to the pre‐ and post‐treatment AMH levels, *gBRCApv* status is a novel potential predictor of amenorrhea at 12 and 18 months after chemotherapy. The higher likelihood of amenorrhea in women *gBRCApv* suggests that they are more prone to losing their fertility post‐chemotherapy.

## Graphical abstract

In a prospective longitudinal analysis in women with breast cancer, we found that pre‐ and post‐chemo AMH and BRCA pathogenic variant status are predictors of post‐chemotherapy amenorrhea risk. This is the first time that an association between BRCA pathogenic variants and amenorrhea risk is established. The information provided in this article can be used in guiding fertility preservation decisions.

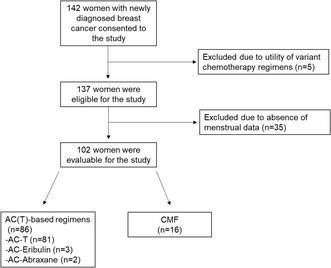

## 1 INTRODUCTION

Breast cancer is the most common cancer worldwide, accounting for 12%–15% of all new cancer cases.^1^ With advanced screening technologies and new therapeutic modalities, the average 5‐year survival rate for women with non‐metastatic invasive breast cancer has reached 90% and mortality declined by 1% per year from 2013 to 2018 and has been steady since then.^2^ Multiple modalities are used in the treatment of breast cancer including cytotoxic chemotherapy. However, most breast cancer chemotherapy regimens contain agents with the long‐term toxic effects of ovarian insufficiency and infertility. Alkylating agents and topoisomerase inhibitors, which form the backbone for most breast cancer regimens cause DNA double strand breaks in ovarian follicles and induce their apoptotic death.^3^ Whereas taxanes do not induce DNA damage in primordial follicles, and therefore, the addition of a taxane to an AC‐based regimen is not expected to compromise ovarian egg reserve,^4^, ^5^ and there have been claims to the contrary based on shorter‐term amenorrhea rates.^6^ The topoisomerase inhibitors can also induce microvascular and stromal damage in the ovary.^7^, ^8^ The sum result of these actions is ovarian insufficiency which often presents with cessation of regular periods.

Several effective fertility preservation techniques have been developed in recent years, which include embryo, oocyte, and ovarian tissue cryopreservation.^9^ While all young women should be counseled on fertility preservation options before initiating chemotherapy, development of tools for post‐chemotherapy amenorrhea risk assessment may help with counseling, decision‐making, and case selection.

To that end, we performed a prospective longitudinal study in young women with breast cancer to determine the predictors of amenorrhea at 12–18 months after the completion of chemotherapy. We specifically investigated age at the onset of chemotherapy, chemotherapy type, tamoxifen use, pre‐ and post‐chemotherapy AMH levels, and *BRCA* pathogenic variant (g*BRCA*pv) status as predictors of amenorrhea at 12–18 months after chemotherapy as temporary amenorrhea is common within the 12 months post‐chemotherapy.^10^

Because *BRCA1* and *2* are DNA DSB repair genes, women with mutations in these genes may have oocytes that are deficient in repairing chemotherapy‐induced damage.^11^, ^12^, ^13^ As chemotherapy causes ovarian follicle death by inducing DNA DSBs, women with g*BRCA*pv may experience greater magnitude of ovarian function loss than the normal population.^13^ We have previously shown that declining *BRCA* function could be critical in ovarian aging^11^, ^12^, ^13^, ^14^ and make ovarian follicle reserve more susceptible to chemotherapy.^13^ For this reason, we also included g*BRCA*pv status as a potential predictor of post‐chemotherapy amenorrhea in breast cancer survivors.

## 2 MATERIALS AND METHODS

This study was approved by the Institutional Review Boards at all participating institutions: Yale University School of Medicine, Memorial Sloan Kettering Cancer Center, and Weill Cornell Medical Center (clinicaltrials.gov identifier NCT00823654). Enrollment began in January 2009, as part of an NIH‐funded translational research project (NICHD and NCI; R01 HD053112) to assess the impact of breast cancer chemotherapy on ovarian reserve and menstrual status and ended in November 2017. Written informed consent was obtained from all patients.

Premenopausal female patients aged 18–44 with breast cancer (Stage I–III), who will receive chemotherapy and with no more than one irregular cycle and/or with at least 10 spontaneous cycles within the past year were included. Prior chemotherapy, hormonal therapy or immunotherapy, previous ovarian surgery, or radiation to pelvic region, plans for risk‐reducing oophorectomy within 1 year of completion of chemotherapy, family history of a first degree relative with non‐surgical menopause before age 40, prior known infertility, and current pregnancy were the exclusion criteria. None of the patients received gonadotropin‐releasing hormone analogs during and after breast cancer treatment.

Amenorrhea was defined as the absence of bleeding for four consecutive menstrual cycles.^15^ Family history was collected at first visit with a medical oncologist, and the National Comprehensive Cancer Network Guidelines were followed for *gBRCApv* testing decision.^16^ At the time of the study period, those with low probability were not tested for *gBRCApv*. These *BRCA*‐untested low risk participants were grouped with those who tested negative for g*BRCA*pv as a single control group, as we previously described.^13^ However, as some women who were previously not tested for g*BRCA*pv based on the older NCNN guidelines later underwent testing, their status was added to the study later on (*n* = 16). None of those who were tested later were g*BRCA*pv‐positive. In addition, we performed a sensitivity analysis excluding those who were not tested for g*BRCA*pv.

AMH levels were measured from frozen‐stored samples that were collected before and after the completion of adjuvant chemotherapy via centralized AMH testing (Ansh Laboratories). Participants kept a menstrual calendar which were reviewed at the completion to determine the menstrual frequency and amenorrhea status at the end of chemotherapy (within 4 weeks of the completion of chemotherapy) and at 6‐, 12‐, and 18‐month time points. We used 12 and 18 months as the primary outcome time points so that we can predict the longer‐term risk of amenorrhea after chemotherapy.

Based on previous data and known information from ovarian biology literature, we investigated age at the onset of chemotherapy, body mass index (BMI), serum AMH levels (pretreatment and immediately after the completion of chemotherapy), chemotherapy type, and g*BRCA*pv status as the possible predictors of menopause in women with invasive breast cancer.^17^

### 2.1 Anti‐Mullerian hormone assay

Sera were aliquoted and stored first at –80°C then long term at −273°C. Frozen aliquots were shipped on dry ice to the laboratory, where serum AMH assay was run on site using an ultra‐sensitive two‐site enzyme‐linked immunoassay (picoAMH ELISA, Ansh Labs). The reportable range was 0.003–23 ng/mL. The coefficient of variation for the four‐levels of pooled serum quality controls tested along with study specimens were reported to be <7%.^10^

### 2.2 Statistical analysis

Descriptive statistics were used to summarize patients' baseline characteristics, including mean ± SD for continuous variables and frequency (%) for categorical variables. We checked possible outlier(s) and its impact on the analyses.

The predictive value of potential predictors for amenorrhea rates at 12 and 18 months was evaluated with simple and multiple regression for unadjusted and multivariable‐adjusted association (with age, BMI, baseline AMH, tamoxifen treatment, chemotherapy regimen, and g*BRCA*pv status as covariates), respectively. From these (non‐longitudinal) logistic regressions, we estimated odds ratio (OR) with 95% confidence interval (CI) and *p*‐value for associational measure between an exposure at baseline and outcome at a follow‐up, with the receiver‐operating‐characteristic (ROC) curve and the associated area of the ROC curve (AUC) for prediction/discrimination measure.^18^ Sensitivity, specificity, and positive and negative predictive values were calculated for binary exposure (e.g., AMH level above/below cutoff) and binary outcome (amenorrhea status at 12 or 18 month), where cutoffs for continuous predictor were suggested by optimal cutoff in ROC curve analyses based on the four different metrics (e.g., Youden index = [Sensitivity + Specificity − 1]; proportion of correct classification; distance from the cutoff to sensitivity = 0 and specificity = 0; and absolute difference between sensitivity and specificity), undetectable threshold (<0.003 ng/mL for post‐treatment AMH), or those used in clinical practice (e.g., age 40, BMI 25). Optimal cutoffs should be considered exploratory (with possible overfit) as not validated in independent datasets. Because AMH is the key ovarian reserve marker, we conducted several exploratory analyses focusing on pre‐ and/or post‐treatment levels. Due to overlapping information and a high proportion of non‐detectability shortly after treatment, we did not include pre‐ and post‐treatment AMH levels together in main regression models.

For longitudinal data analyses that analyzed repeated outcome measures (0, 6, 12, 18 months together), we used a generalized estimating equation approach that accounts for correlation within person (namely, population‐averaged model). In this model, the interaction effect of time since baseline and *BRCA* status served as our primary parameter of interest, where this interaction term estimates “time trend” (or difference in slopes; the ratio of odds ratio [ROR]) in the amenorrhea rate/probability for *BRCA* positive versus not groups,^19^, ^20^ while adjusting other covariates (age, BMI, AMH, Tamoxifen, and regimen) as dichotomized variable/group (as high vs. low, Y vs. N) to test the difference in time trend between each comparison group via time*group interaction. Finally, we conducted sensitivity analyses to assess the robustness of our key finding on *BRCA*: (1) accounting for time trend only, not adjusting for other covariates; (2) using a different cutoff for AMH or age; (3) using a generalized linear mixed model (namely, subject‐specific model)^17^; and (4) repeating our analyses after excluding 21 women who did not undergo g*BRCA*pv testing. Statistical analyses were performed with SAS 9.4.

## 3 RESULTS

### 3.1 Description of the study population

**FIGURE 1:**
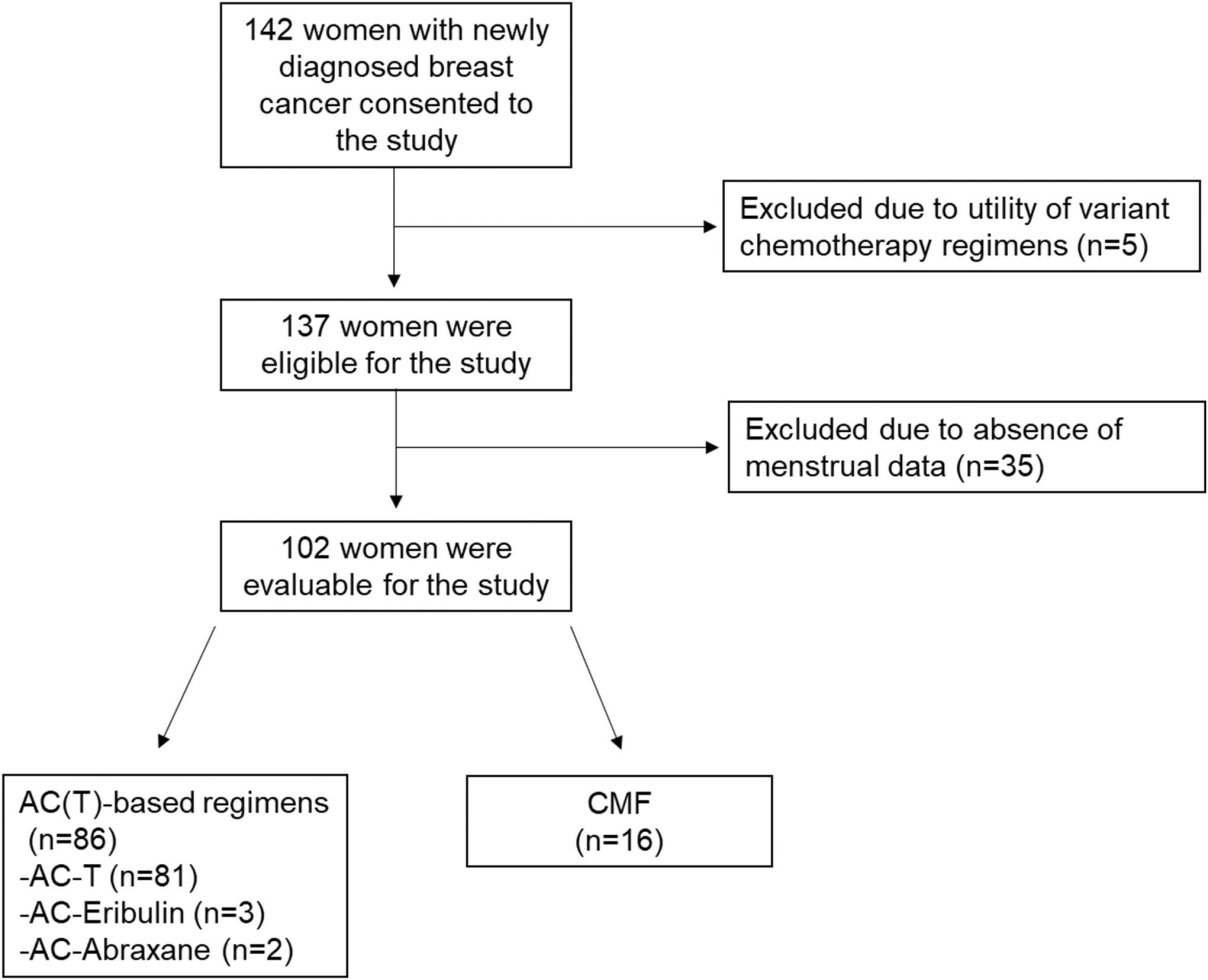
Inclusion–exclusion flow chart. AC(T), Antracycline‐cyclophosphamide (Taxane); CMF, Cyclophosphamide‐Methotrexate +5‐Fluorouracil.

A total of 142 premenopausal women with newly diagnosed breast cancer were enrolled. Of those who received adjuvant chemotherapy, 5 were excluded due to the utility of variant chemotherapy regimens. From the remaining, 35 were excluded as they were not evaluable because they did not keep a menstrual calendar. Finally, 102 women with a mean age of 37.4 ± 4.8 years who received an AC‐based (Anthracycline‐Cyclophosphamide followed by taxanes in >90% of patients; *n* = 86) or CMF (Cyclophosphamide‐Methotrexate +5‐Fluorouracil; *n* = 16) regimen were evaluable (Figure 1; Table 1). Twenty‐three patients had HER2+ disease. Of these 23 women, all patients received HER2 targeting therapy (Herceptin). Out of 102 study patients, 12 carried a pathogenic variant of *BRCA*1/2 and the remaining either tested negative (*n* = 67) or were not tested (*n* = 23). The mean age of women with g*BRCA*pv was 34.3 ± 4.5 years, lower than those without g*BRCA*pv or untested 37.7 ± 4.7 (*p* = 0.03). Amenorrhea rates declined from 6 to 12 months (51% vs. 38%, *p* = 0.009, McNemar test), with a less apparent change from 12 to 18 months after the completion of chemotherapy (38% vs. 31%, *p* = 0.05). Subjects were made aware at enrollment that oophorectomies while on study would make them ineligible; most women expressed interest in maintaining their fertility after treatment.

**TABLE 1:** Baseline characteristics of evaluable study participants (*n* = 102).

Variables	mean ± SD or *n* (%)
Age (years)	37.4 ± 4.8
Body mass index (kg/m^2^)^a^	24.4 ± 4.7
Stage	
I	30 (29.3)
II	49 (48.0)
III	23 (22.6)
Estrogen receptor status	
Positive	84 (82.4)
Negative	18 (17.6)
Progesteron receptor status	
Positive	82 (80.4)
Negative	20 (19.6)
HER2 status	
Positive	24 (23.5)
Negative	78 (76.5)
*gBRCApv* status	
Positive	12 (11.8)
Negative	69 (67.6)
Untested	21 (20.5)
Tamoxifen use (following chemotherapy)	
Yes	81 (80.2)
No	20 (19.8)
Treatment regimen	
AC‐based	86 (84.3)
AC‐T	81 (94.1)
AC‐Eribulin^b^	3 (3.4)
AC‐Abraxane	2 (2.3)
CMF	16 (15.7)
AMH (ng/mL)	3.06 ± 2.62 Median = 2.50; IQR: 1.16–4.20

Abbreviations: AC, Anthracycline‐Cyclophosphamide; AMH, anti‐mullerian hormone; CMF, Cyclophosphamide‐Methotrexate +5‐Fluorouracil; *gBRCAp*v, germline BRCA pathogenic variant; IQR, interquartile range; T, Taxane; SD, standard deviation.

^a^ 1 missing value, therefore *n* = 101.

^b^ Patients were on Eribulin as part of another trial and were still being treated with Doxorubicin.

### 3.2 AMH thresholds for amenorrhea risk

Based on the ROC curve analysis with baseline AMH as predictor and 12‐month amenorrhea, optimal cutoff of ~1.7 ng/mL was suggested by Youden index and correct classification, and ~2.0 ng/mL was suggested by the two other measures. For amenorrhea at 18 months, ~2.0 ng/mL was further suggested by Youden index. Thus, we selected 2.0 ng/mL as our primary cutoff when analyzing AMH as a binary variable/predictor. Results were presented in Figure S1 and Table S1.

Age of 40 years has been commonly used as a threshold for increased amenorrhea risks^21^, ^22^ but there has not been a study to test this assumption. While we did not perform this study to test this assumption, we opportunistically explored optimal age cutoff values with ROC curve analysis and found that age thresholds of 39–44 yielded highest accuracy in the prediction of the risk of amenorrhea using the four different metrics (listed in the Section 2.2); for example, 43 was selected by Youden index at 12 and 18 months. This suggests that, while an age cutoff point of around 40 years is possible, further studies maybe needed to determine a more precise age threshold.

### 3.3 Predictive value of AMH for amenorrhea risk

The discriminative and predictive abilities of AMH in the prediction of amenorrhea at different time points in all patients were assessed (Table S2). The pretreatment AMH level (as a continuous predictor alone) significantly predicted amenorrhea at 12 and 18 months (*p* = 0.004, AUC 0.74 and *p* = 0.01, AUC 0.73, respectively), whereas post‐chemo AMH (detectable vs. not) predicted amenorrhea at 12 months (*p* = 0.01, AUC 0.68) and at 6 months (*p* = 0.0001, AUC 0.82). When we used the pre‐ and post‐chemo AMH together as predictors, the AUC was increased to 0.80 for the 12 months, but not the 18 months.

Predictive performance of AMH for amenorrhea at baseline versus post‐chemotherapy was also shown in Table S1. For example, AMH at baseline (high vs. low based on 2.0 ng/mL cutoff) yielded sensitivity of 66% and specificity of 70% at 12 months, and sensitivity of 70% and specificity of 69% at 18 months. The lower cutoffs naturally reduce sensitivity but enhanced specificity. In contrast, undetectable versus detectable AMH levels post‐chemotherapy showed better predictive performance for shorter follow‐up; best for 6 months (sensitivity 86% and specificity 71%) and reduced performance for 12 and 18 months. Most analyses in Table S1 yielded positive predictive value >0.5, which implies that over 50% of patients who had an AMH below the given threshold are expected to experience amenorrhea.

### 3.4 The predictors of post‐chemotherapy amenorrhea via regression analyses

We performed regression analyses for a binary outcome (amenorrhea vs. no amenorrhea) with the 6 predictors via two different approaches to identify baseline factors that are associated with: (1) the amenorrhea at 12 or 18 months separately, and (2) time trend over 0, 6, 12, and 18 months together. We refer to the former analysis as “non‐longitudinal” logistic regression, where OR serves as an associational measure (between predictor and outcome). We refer to the latter as “longitudinal” regression counterparts that estimate and test the difference in the time trend of the event rate between the comparison groups defined by each of the six factors, where ROR serves as a relative associational measure.

In this multivariable‐adjusted logistic regression, age and baseline AMH were significantly associated with the outcome (*p* = 0.03) at 12 months; these associations did not reach statistical significance (at *p* = 0.05 level) at 18 months (*p* = 0.07–0.08). In contrast, a *gBRCApv* was found to be a significant predictor for amenorrhea at 18 months post‐chemotherapy (OR = 5.59 [95% CI = 1.21, 25.8], *p* = 0.03). Although statistical significance was not reached for the 12‐month time point, the point and interval estimates showed a positive association (OR = 2.15 [95% CI = 0.52, 8.89]) (Table 2).

**TABLE 2:** Unadjusted and multivariable‐adjusted logistic regression for amenorrhea at 6, 12, and 18 months with predictors at baseline.

Predictors	Unadjusted model odds ratio (95% CI), *p*‐value	Adjusted model odds ratio (95% CI), *p*‐value AUC = 0.80
(a) Outcome = amenorrhea at 6 months		
AMH at baseline (*n* = 102) (per 0.1 increase)	0.98 (0.97, 1.00), *p* = 0.04	0.98 (0.96, 1.00), *p* = 0.12
Age (per 1 year) (*N* = 102)	1.10 (1.01, 1.20), *p* = 0.03	1.18 (1.05, 1.34), *p* = 0.006
BMI (per 1 unit) (*n* = 102)	1.09 (1.00, 1.20), *p* = 0.06	1.04 (0.93, 1.16), *p* = 0.49
Tamoxifen (*n* = 81)	0.29 (0.10, 0.87), *p* = 0.03	0.31 (0.09, 1.11), *p* = 0.07
AC‐based regimen (*n* = 86)	5.90 (1.56, 22.2), *p* = 0.009	13.4 (2.85, 63.2), *p* = 0.001
*gBRCApv*‐positive (*n* = 12)	0.76 (0.22, 2.68), *p* = 0.67	0.90 (0.22, 3.66), *p* = 0.88

Predictors	Unadjusted model odds ratio (95% CI), *p*‐value	Adjusted model odds ratio (95% CI), *p*‐value AUC = 0.77
(b) Outcome = amenorrhea at 12 months		
AMH at baseline (*n* = 102) (per 0.1 increase)	0.96 (0.94, 0.99), *p* = 0.004	0.97 (0.95, 1.00), *p* = 0.03
Age (per 1 year) (*n* = 102)	1.13 (1.04, 1.25), *p* = 0.008	1.15 (1.02, 1.30), *p* = 0.03
BMI (per 1 unit) (*n* = 102)	1.09 (1.00, 1.19), *p* = 0.05	1.07 (0.97, 1.18), *p* = 0.17
Tamoxifen (*n* = 81)	0.90 (0.33, 2.45), *p* = 0.84	1.00 (0.31, 3.25), *p* = 0.99
AC‐based regimen (*n* = 86)	1.40 (0.45, 4.38), *p* = 0.57	2.88 (0.73, 11.4), *p* = 0.13
*gBRCApv*‐positive (*n* = 12)	1.44 (0.41, 5.08), *p* = 0.57	2.15 (0.52, 8.89), *p* = 0.29

Predictors	Unadjusted model odds ratio (95% CI), *p*‐value	Adjusted model odds ratio (95% CI), *p*‐value AUC = 0.76
(c) Outcome = amenorrhea at 18 months		
AMH at baseline (*n* = 102) (per 0.1 increase)	0.97 (0.94, 0.99), *p* = 0.01	0.98 (0.95, 1.00), *p* = 0.07
Age (per 1 year) (*n* = 102)	1.12 (1.02, 1.24), *p* = 0.02	1.12 (0.99, 1.27), *p* = 0.08
BMI (per 1 unit) (*n* = 102)	1.03 (0.94, 1.12), *p* = 0.54	1.04 (0.93, 1.16), *p* = 0.49
Tamoxifen (*n* = 81)	2.42 (0.64, 9.17), *p* = 0.19	2.64 (0.56, 12.3), *p* = 0.22
AC‐based regimen (*n* = 86)	0.70 (0.23, 2.15), *p* = 0.53	1.31 (0.34, 5.01), *p* = 0.69
*gBRCApv*‐positive (*n* = 12)	3.10 (0.87, 11.1), *p* = 0.08	5.59 (1.21, 25.8), *p* = 0.03

*Note*: AUC denotes area under ROC curve derived from logistic regression, a discrimination statistic.

Abbreviations: AMH, anti‐mullerian hormone; BMI, body mass index; CMF, Cyclophosphamide‐Methotrexate +5‐Fluorouracil; *gBRCApv*, germline *BRCA* pathogenic variant.

Finally, longitudinal analyses confirmed a beneficial effect of high baseline AMH levels on the risk of amenorrhea during the follow‐up (ROR = 0.91 [95% CI = 0.86–0.97], *p* = 0.002) for differential time trends (per month) in the event rate for high versus low (> vs. ≤2.0 ng/mL) AMH groups. In addition, the g*BRCApv*‐positive group showed a steeper slope in the OR of amenorrhea: 12% higher OR versus the non‐positive group (ROR = 1.12 [95% CI = 1.04–1.20], *p* = 0.003) (Table 3). Other comparison groups (defined by age, BMI, tamoxifen treatment, and type of chemotherapy regimen) showed from 2% decrease to 5% increases in OR with *p*‐values >0.09. Our sensitivity analyses demonstrated the robustness of these findings, for example, yielding 8%–10% increased odds of amenorrhea for g*BRCA*pv carriers, with *p*‐values of 0.006–0.03 (Table S3). In our sensitivity analyses after the exclusion of patients whose g*BRCApv* status was unknown, we reached stronger associations despite the reduced sample size. For example, we obtained an unadjusted OR of 3.53 (*p* = 0.06) and an adjusted OR of 6.03 (*p* = 0.02) in Table 2C, as well as an ROR of 1.12 (*p* = 0.002) in Table 3 for the g*BRCApv*‐positive group (Tables S4 and S5).

**TABLE 3:** Longitudinal analysis at 0, 6, 12, and 18 months for the difference in amenorrhea trend between groups dichotomized by baseline factors.

Difference in time trend between 2 groups (per 1 month)	Multivariables‐adjusted model ratio of odds ratios (95% CI), *p*‐value
Reference group^a^	0.98 (0.89, 1.08), *p* = 0.65
AMH at baseline: > vs. ≤2.0^b^ (*n* = 102)	0.91 (0.86, 0.97), *p* = 0.002
Age: > vs. ≤40 (*n* = 102)	1.05 (0.99, 1.11), *p* = 0.14
BMI: > vs. ≤25 (*n* = 102)	1.05 (0.99, 1.11), *p* = 0.09
Tamoxifen (*n* = 81) vs. not	1.05 (0.96, 1.14), *p* = 0.31
AC‐based (*n* = 86) vs. CMF regimen (*n* = 16)	0.98 (0.90, 1.06), *p* = 0.57
*gBRCApv* (*n* = 12) vs. not (*n* = 67)	1.12 (1.04, 1.20), *p* = 0.003

*Note*: Ratio of odds ratio = 1 indicates null value, that is, no difference in time trend between 2 groups, measured by odds ratio (per 1 month increase and outcome) in each group separately. Longitudinal data were fit via a generalized estimating equation. Sensitivity analyses (e.g., based on generalized linear mixed effects model) are presented in Table S3.

Abbreviations: AC, Anthracycline‐Cyclophosphamide based regimen, >90% with a taxane; AMH, Anti‐mullerian hormone; BMI, body mass index; CMF, Cyclophosphamide‐Methotrexate +5‐Fluorouracil; *gBRCApv*, germline BRCA pathogenic variant.

^a^ Time trend (i.e., odds ratio for month) for reference group of AMH ≤ 2.0, Age ≤ 40, BMI ≤ 25, no tamoxifen, AC‐based and not g*BRCApv*‐positive.

^b^ Cutoff was suggested from ROC curve in Figure S1.

## 4 DISCUSSION

In this prospective longitudinal study, we studied the predictors of amenorrhea risk at a delayed time range of 12–18 months after the last dose of breast cancer chemotherapy. We evaluated the predictive capability of variables including age, pre‐ and post‐chemotherapy AMH levels, tamoxifen use, and g*BRCA*pv status for amenorrhea status at 12 and 18 months. We found that age at the onset of treatment as previously known, pre‐ and post‐treatment AMH levels, and as a novel finding, g*BRCA*pv status can be predictors of amenorrhea at 12–18 months.

We found three studies that explored the predictors of amenorrhea such as age, pre‐ and post‐treatment AMH as well as tamoxifen treatment after breast cancer chemotherapy.^23^, ^24^, ^25^ All three studies looked at the value of AMH in prediction of amenorrhea risk, but none with the incorporation of g*BRCA*pv status as a predictor.^23^, ^24^, ^25^ In those studies, even though the samples were collected longitudinally, the authors performed standard association or survival analysis, not studying time trends over longitudinal data. In two studies by the same group,^23^, ^24^ the maximum follow‐up was 2 years after the diagnosis, which would have corresponded to <18 months follow‐up from the completion of chemotherapy. In the first study in 2013, there were 55 and 46 women evaluable at 12 and 24 months after the breast cancer diagnosis, respectively, and the authors found an AUC for pretreatment AMH of 0.90 (95% CI 0.82–0.97), and for age, 0.88 (95% CI 0.78–0.97). In the second report where the data from OPTION study (*n* = 101) were analyzed secondarily, data from women who were not treated with GnRHa (*n* = 22) were analyzed to assess the value of AMH measured after the end of chemotherapy for prediction of amenorrhea at 24 months and the AUC was 0.84. The study found an AMH cutoff of 7.3 pmol/L (1.06 ng/mL) for predicting POI (AUC 0.77) at 24 months after the diagnosis. In both reports, various chemotherapy protocols were utilized, making it difficult to pin the amenorrhea risk to a specific regimen. In the third study,^25^ 82 participants from the secondary analysis of ASTRRA trial were included to evaluate the predictive value of AMH within 2 months of the final dose of chemotherapy. On multivariate analysis, post‐chemotherapy AMH (hazard ratio: 2.85, 95% CI: 1.01–8.05, *p* = 0.048) was found to be a significant independent predictor for resumption of menstruation after a 5‐year follow‐up.

To our knowledge, our study is the first and largest to offer longitudinal analyses, which allowed multivariable adjustments including g*BRCA*pv status and chemotherapy type, and associations as well as time trends (up to 18 months) to be studied. Moreover, our study population received two main chemotherapy regimens, predominantly anthracycline and taxane based, making the analysis of the impact of chemotherapy on amenorrhea risk more homogenous. Particularly, the impact of g*BRCA*pv status on amenorrhea rates was not assessed in the prediction models of previous studies and we showed for the first time that women with g*BRCA*pv appear to be more prone to losing their fertility post‐chemotherapy.

We found that the presence of a g*BRCA*pv strongly predicted the amenorrhea risk at 18 months with likely progression of the event rate during 0–18 months after the completion of post‐chemotherapy. Despite the limited size of the *BRCA*‐positive subgroup, the associations related to AMH levels and g*BRCA*pv appeared to be robust over different analytic strategies (i.e., unadjusted and adjusted analyses, non‐longitudinal and longitudinal analyses, population‐averaged and subject‐specific models, and various sensitivity analyses). When we limited the analysis to women whose gBRCApv status was known, we reached stronger associations despite the reduced sample size (Tables S4 and S5).

The association of g*BRCA*pv with amenorrhea risk is biologically plausible as *BRCA* genes play essential roles in ATM‐mediated repair of DNA double strand breaks.^14^ We have previously shown that the oocytes of women with g*BRCA*pv are deficient in DNA repair and subject to accelerated age‐induced loss.^11^, ^12^ Because both cyclophosphamide and doxorubicin cause ovarian follicle death by inducing DNA DSBs in oocytes, the deficient DNA repair mechanisms in oocytes of women with g*BRCA*pv may result in increased liability to chemotherapy‐induced ovarian damage.^7^, ^12^ In support of this hypothesis, we have in multiple studies including a recent large multi‐center individual patient data meta‐analysis that women with g*BRCApv* had lower serum AMH recovery after chemotherapy compared to controls.^12^, ^26^ While numerous studies confirmed our findings, few others failed to detect the impact of BRCA mutations on ovarian reserve, methodological weaknesses of which were discussed in a recent review.^14^ Lower baseline serum AMH levels, and hence a lower ovarian reserve in women with gBRCApv may have also contributed to the predisposition to amenorrhea risk. However, in mechanistic support of our findings, we also found that when *BRCA1* was knocked out in mouse oocytes, they became more liable to doxorubicin‐induced death in vitro.^12^, ^13^ Moreover, in a previous study we found that women with g*BRCA*pv had slower AMH recovery post‐chemotherapy compared to those who were negative or untested for g*BRCA*pv.^13^ The stronger prediction of amenorrhea at the 18‐month time point and clear monotonic positive trend may also be explained by the fact that the follicle loss could be further accelerated in the ovaries of women with *BRCA* mutations with advancing age.^12^ Women with gBRCApv may face additional difficulties when they try to conceive such as, for some, the need for preimplantation genetic diagnosis through IVF to prevent the transmission of g*BRCA*pv to their offspring, and reduced reproductive lifespan due to risk‐reducing salpingo oophorectomy. Therefore, fertility preservation with embryo or oocyte cryopreservation can be options for those women, followed by for preimplantation genetic testing if they consent.

The number of women with BRCA mutations is relatively limited in our cohort. However, given the longitudinal nature of our study the associations are robust. Based on our findings, we suggest that women with baseline AMH of <2.0 ng/mL, undetectable AMH post‐chemotherapy, and who have g*BRCA*pv may be prioritized for fertility preservation counseling before AC‐based (with a taxane) or CMF regimens. Although not as commonly utilized in all parts of the world, CMF is still administered in institutions that participated in this study, and in other major cancer centers in Northeastern USA. Moreover, given that the gonadotoxic component in CMF is cyclophosphamide, we believe that the information obtained with this protocol is still useful in showing that the ovaries of women with g*BRCApv* are more liable to gonadotoxic cancer treatments.

Twenty‐four percent of the participants in this study were HER2‐positive. Although the use of trastuzumab and other anti‐HER2 treatments do not seem to increase the risk of treatment‐related amenorrhea, further research will be needed to investigate their gonadotoxic potential, if any.^27^

With the aid of prediction methods developed based on the data presented here, patients with higher risk of post‐chemotherapy ovarian insufficiency can be offered early counseling and fertility preservation action. That may then enable consecutive ovarian stimulation cycles with aromatase inhibitors to increase the likelihood of success with fertility preservation with oocyte or embryo cryopreservation.^28^, ^29^ One may also combine oocyte and embryo cryopreservation procedures with ovarian tissue cryopreservation to preserve ovarian function, as the latter is no longer considered an experimental approach.^30^, ^31^, ^32^

In conclusion, in addition to pre‐ and post‐chemotherapy AMH levels, g*BRCA*pv status can be a potential predictor of amenorrhea at 12–18 months post‐chemotherapy. The higher likelihood of amenorrhea in women with g*BRCA*pv indicate that these women may be more prone to losing their fertility post‐chemotherapy, and that they should be accordingly counseled. However, the sample size of women with g*BRCA*pv was small in our study. Our findings may help cancer practitioners in patient selection with fertility preservation referrals, and patients in making better informed decisions when considering these procedures. Future larger prospective longitudinal studies may provide more precise information and external validation on the association of g*BRCA*pv and the amenorrhea risk, potentially leading to the development of a rigorous prediction model.

## AUTHOR CONTRIBUTIONS

**Kutluk H. Oktay:** Conceptualization (lead); funding acquisition (lead); methodology (lead); project administration (lead); validation (lead); writing – original draft (lead); writing – review and editing (lead). **Volkan Turan:** Writing – original draft (supporting); writing – review and editing (supporting). **Giuliano Bedoschi:** Data curation (supporting); resources (supporting); visualization (supporting). **Nadia Abdo:** Data curation (equal); investigation (equal); supervision (supporting). **Heejung Bang:** Formal analysis (lead); software (lead); writing – original draft (equal); writing – review and editing (equal). **Shari Goldfarb:** Data curation (equal); investigation (equal); methodology (equal); project administration (equal); resources (equal); supervision (equal); validation (equal); writing – review and editing (equal).

## FUNDING INFORMATION

This study was supported by RO1 HD053112 from the Eunice Kennedy Shriver National Institute of Child Health and Human Development (NICHD), and National Cancer Institute. HB is partly supported by the National Institutes of Health through grant UL1 TR001860.

## CONFLICT OF INTEREST STATEMENT

The authors declare no conflict of interest.

## Supporting information


Data S1:

## Data Availability

The data that support the findings of this study are available on request from the corresponding author. The data are not publicly available due to privacy or ethical restrictions.
