# Supplementary material for: A prospective longitudinal analysis of the predictors of amenorrhea after breast cancer chemotherapy: Impact of BRCA pathogenic variants
Source: Cancer Med. 2023 Sep 12;12(18):19225–33. doi: 10.1002/cam4.6527 (PMC10557848; doi:10.1002/cam4.6527)

**SUPPLEMENT**

Table S1. Prediction performance of AMH for amenorrhea at 6, 12 and 18 months

| Predictor | AMH cut-off  (ng/mL) | Amenorrheic  @ month: | Sensitivity | Specificity | PPV | NPV |
| --- | --- | --- | --- | --- | --- | --- |
| AMH at  Baseline  (n=102) | 1.7* | 6 | 44 | 76 | 66 | 56 |
|  |  | 12 | 58 | 79 | 63 | 76 |
|  |  | 18 | 60 | 78 | 55 | 81 |
|  | 2.0* | 6 | 52 | 65 | 61 | 56 |
|  |  | 12 | 66 | 70 | 57 | 77 |
|  |  | 18 | 70 | 69 | 50 | 84 |
| AMH at the end of chemotherapy  (n=96) | 0.003** | 6 | 86 | 71 | 77 | 82 |
|  |  | 12 | 78 | 52 | 51 | 79 |
|  |  | 18 | 69 | 48 | 38 | 77 |

*AMH cut-offs were derived from ROC curve in Figure S1.

**Detection limit-undetectable; 57 values were <0.003 ng/mL and 6 were missing among 102 patients.

PPV: positive predictive value; NPV: negative predictive value.

Table S2. Discrimination ability of AMH in the prediction of amenorrhea at 6, 12 and 18 months after the completion of chemotherapy

| Outcome =  Amenorrhea | Predictors in logistic regression  (p-value) | AUC |
| --- | --- | --- |
| At 6 months  (n=101) | Age (p=0.03) plus  BMI at baseline (p=0.06) | 0.67 |
|  | AMH at baseline (p=0.04) | 0.66 |
|  | AMH at the end of chemo (p=0.0001) | 0.82 |
|  | AMH at baseline (p=0.18) plus  AMH at the end of chemo (p=0.0002) | 0.86 |
| At 12 months  (n=101) | Age (p=0.008) plus  BMI at baseline (p=0.05) | 0.68 |
|  | AMH at baseline (p=0.004) | 0.74 |
|  | AMH at the end of chemo (p=0.01) | 0.68 |
|  | AMH at baseline (p=0.02) plus  AMH at the end of chemo (p=0.03) | 0.80 |
| At 18 months  (n=97) | Age (p=0.03) plus  BMI at baseline (p=0.55) | 0.64 |
|  | AMH at baseline (p=0.01) | 0.73 |
|  | AMH at the end of chemo (p=0.12) | 0.60 |
|  | AMH at baseline (p=0.03) plus  AMH at the end of chemo (p=0.28) | 0.72 |

AUC denotes area under ROC curve derived from logistic regression, a discrimination statistic.

Table S3. Sample sensitivity analyses of Table 3

a) Unadjusted model

| Difference in time trend between 2 groups (per 1 month) | Multivariables-adjusted model  Ratio of Odds Ratios (95% CI), p-value |
| --- | --- |
| Reference group+ | 0.99 (0.96, 1.03), p=0.67 |
| *gBRCApv-*positive (n=12) vs. not (n=90) | 1.08 (1.01, 1.16), p=0.03 |

b) Using a different cut-off for AMH

| Difference in time trend between 2 groups (per 1 month) | Multivariables-adjusted model  Ratio of Odds Ratios (95% CI), p-value |
| --- | --- |
| Reference group+ | 0.98 (0.88, 1.08), p=0.65 |
| AMH at baseline > vs. ≤1.7* | 0.92 (0.87, 0.97), p=0.004 |
| Age > vs. ≤40* | 1.04 (0.98, 1.11), p=0.17 |
| BMI > vs. ≤25 | 1.04 (0.99, 1.10), p=0.12 |
| Tamoxifen (n=81) vs. not | 1.05 (0.96, 1.15), p=0.25 |
| AC-based (n=86) vs CMF regimen (n=16) | 0.99 (0.91, 1.07), p=0.73 |
| *gBRCApv-*positive (n=12) vs. not (n=90) | 1.10 (1.03, 1.18), p=0.006 |

c) Using a different cut-off for age

| Difference in time trend between 2 groups (per 1 month) | Multivariables-adjusted model  Ratio of Odds Ratios (95% CI), p-value |
| --- | --- |
| Reference group+ | 0.98 (0.89, 1.08), p=0.68 |
| AMH at baseline: > vs. ≤2.0* | 0.93 (0.87, 0.99), p=0.02 |
| Age: > vs. ≤43* | 1.08 (0.99, 1.18), p=0.09 |
| BMI: > vs. ≤25 | 1.05 (0.99, 1.11), p=0.09 |
| Tamoxifen (n=81) vs. not | 1.04 (0.96, 1.13), p=0.31 |
| AC-based (n=86) vs. CMF regimen | 0.99 (0.91, 1.07), p=0.78 |
| *gBRCApv* positive (n=12) vs. not (n=90) | 1.09 (1.02, 1.17), p=0.01 |

d) Generalized linear mixed effect model

| Difference in time trend between 2 groups (per 1 month) | Multivariables-adjusted model  Ratio of Odds Ratios (95% CI), p-value |
| --- | --- |
| Reference group+ | 1.03 (0.92, 1.15), p=0.66 |
| AMH at baseline: > vs. ≤2.0* | 0.89 (0.85, 0.94), p<0.0001 |
| Age: > vs. ≤40 | 1.09 (1.02, 1.16), p=0.01 |
| BMI: > vs. ≤25 | 1.03 (0.98, 1.09), p=0.27 |
| Tamoxifen (n=81) vs. not | 1.00 (0.93, 1.06), p=0.90 |
| AC-based (n=86) vs. CMF regimen | 0.93 (0.85, 1.02), p=0.11 |
| *gBRCApv*-positive (n=12) vs. not (n=90) | 1.10 (1.01, 1.19), p=0.02 |

+Time trend (i.e., odds ratio for month) for Reference group; e.g. not *gBRCApv*-positive for a) and AMH≤1.7, Age≤40, BMI≤25, no tamoxifen, AC-based and not *gBRCApv* -positive for b).

*Sample cutoffs were suggested from ROC curve in Figure S1.

When we adjusted the main effect of *gBRCApv* in the model (a) above, we reached p=0.01 for *gBRCApv*  group’s time trend.

Table S4. Multivariable-adjusted logistic regression for amenorrhea at 6, 12, 18 months with predictors at baseline, after excluding patients with *BRCA* status not tested

| Predictors | Adjusted Odds Ratio (95% CI), p-value | | |
| --- | --- | --- | --- |
|  | 6 months  (AUC=0.77), n=78 | 12 months  (AUC=0.78), n=79 | 18 months (AUC=0.77), n=76 |
| AMH at baseline  (per 0.1 increase) | 0.99 (0.97, 1.01), p=0.24 | 0.97 (0.94, 1.00), p=0.03 | 0.97 (0.94, 1.01), p=0.10 |
| Age (per 1 year) | 1.16 (1.02, 1.33), p=0.03 | 1.16 (1.01, 1.33), p=0.04 | 1.12 (0.97, 1.30), p=0.11 |
| BMI (per 1 unit) | 1.06 (0.95, 1.19), p=0.31 | 1.05 (0.94, 1.17), p=0.40 | 1.03 (0.91, 1.15), p=0.68 |
| Tamoxifen | 0.59 (0.15, 2.32), p=0.45 | 1.08 (0.26, 4.46), p=0.92 | 2.99 (0.47, 19.1), p=0.25 |
| AC-based regimen | 13.9 (2.18, 100), p=0.005 | 4.76 (0.87, 26.3), p=0.07 | 2.82 (0.50, 15.9), p=0.24 |
| *gBRCApv*-positive | 0.87 (0.21, 3.54), p=0.84 | 2.05 (0.48, 8.74), p=0.33 | 6.03 (1.26, 28.9), p=0.02 |

This table is same as Table 2’ right columns after excluding the patients without *BRCA* test results.

Table S5. Longitudinal analysis at 0, 6, 12 and 18 months for the difference in amenorrhea trend between groups dichotomized by baseline factors, after excluding patients with *BRCA* status not tested

| Difference in time trend between 2 groups (per 1 month) | Multivariables-adjusted model  Ratio of Odds Ratios (95% CI), p-value |
| --- | --- |
| Reference group | 1.01 (0.93, 1.10), p=0.79 |
| AMH at baseline: > vs. ≤2.0 | 0.89 (0.83, 0.96), p=0.001 |
| Age: > vs. ≤40 | 1.05 (0.97, 1.13), p=0.23 |
| BMI: > vs. ≤25 | 1.04 (0.98, 1.11), p=0.23 |
| Tamoxifen vs. not | 1.03 (0.95, 1.12), p=0.43 |
| AC-based vs CMF regimen | 0.93 (0.84, 1.03), p=0.16 |
| *gBRCApv-positive* vs. not | 1.12 (1.04, 1.21), p=0.002 |

This table is same as Table 3 after excluding the patients without BRCA test results.

Figure S1. ROC curve with continuous predictors, AMH at baseline (upper), age (middle) or BMI (lower), as a sole predictor for amenorrhea at 12 months (n=101)


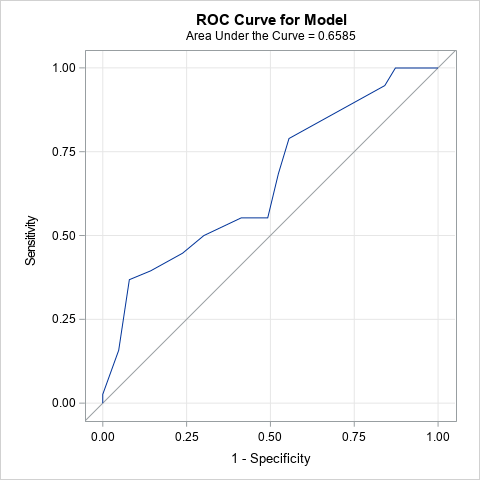


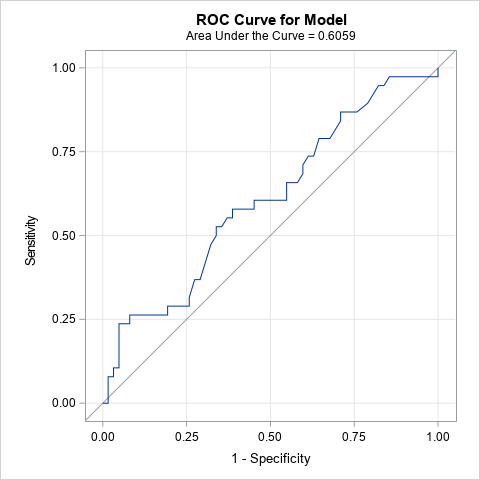

Supplement: Supplementary file 1 — Data S1: [file CAM4-12-19225-s001.docx]
